# Unveiling the Multilevel Drivers of Presenteeism in Emergency Nursing: A Qualitative Inquiry Into Health, Work, and Organizational Dynamics

**DOI:** 10.1155/jonm/5021276

**Published:** 2026-08-04

**Authors:** Zhuojing Yang, Li Niu, Yuyan Zhao, Xiaoyi Cao, Lili Wang, Qian Zhao, Juzi Wang

**Affiliations:** ^1^ Department of Nursing, Shanxi Provincial People’s Hospital Affiliated to Shanxi Medical University, Taiyuan, Shanxi, China; ^2^ School of Nursing, Shanxi Medical University, Taiyuan, Shanxi, China, sxmu.edu.cn; ^3^ School of Nursing, Shanxi University of Chinese Medicine, Jinzhong, Shanxi, China, sxtcm.com

**Keywords:** emergency department, influencing factors, nurse, presenteeism, qualitative research

## Abstract

**Objective:**

This study seeks to identify the factors influencing presenteeism among emergency nurses to inform the development of strategies aimed at reducing presenteeism in the emergency department setting.

**Methods:**

This study adopted a qualitative design informed by a descriptive phenomenological perspective. Purposive sampling was used to recruit 15 emergency nurses from a tertiary hospital for semistructured, face‐to‐face, in‐depth interviews. The interview guide was informed by the theoretical framework of presenteeism behavior, and data were analyzed using Colaizzi’s seven‐step phenomenological analysis method.

**Results:**

Our research identified that presenteeism among emergency nurses is influenced by personal health factors, including physical and psychological well‐being, work‐related factors such as job satisfaction, burnout, and job performance, and social and organizational support factors, including family support, collegial and team support, and institutional policies. The analysis culminated in the identification of three main themes and eight subthemes.

**Conclusion:**

Emergency nurses exhibit a positive professional stance and employ strategic responses to presenteeism. Multilevel interventions involving individual engagement, social support, collegial and team support, and organizational policies may help mitigate presenteeism, enhance nursing performance and satisfaction, and consequently elevate the overall quality of nursing care and patient safety.

**Summary**
• Implications for nursing management.
◦ Nurse managers should recognize presenteeism among emergency nurses as a multilevel workforce management issue shaped by personal health, work‐related demands, social support, team functioning, and institutional policies. Management strategies should include establishing a nonpunitive sick‐leave culture, optimizing flexible staffing and rostering, providing timely workload redistribution, strengthening collegial and team support, and offering accessible psychological and health‐support resources. These measures may help emergency nurses make safer work‐participation decisions, reduce burnout and presenteeism, enhance job satisfaction and performance, and ultimately protect patient safety and care quality.
• Patient or public contributions
◦ This study did not include patient or public involvement in its design, conduct or reporting.

## 1. Introduction

Emergency department (ED) nurses provide care in a clinical setting where patient conditions can change rapidly, and workload is often difficult to predict [1, 2]. In this context, adequate staffing, continuity of care, and timely clinical response are closely linked to patient safety and the efficient operation of the ED [3, 4]. ED nurses are expected to manage acute changes in patients’ conditions, carry out urgent nursing interventions, communicate with multiple professional teams, and maintain team coordination under considerable workload and emotional pressure. When staffing is limited, patient volume is high, or taking sick leave may place additional pressure on colleagues, some nurses may choose to remain at work despite physical discomfort or psychological distress.

Presenteeism, defined as “the behavior of attending work despite ill health when rest would be medically appropriate,” has been extensively studied across various professions [5–7]. ED nurses frequently work while unwell due to the demanding nature of their jobs, which not only jeopardizes their physical and mental well‐being but also compromises care quality and patient safety [8–10]. A deeper understanding of the factors influencing presenteeism in this population is critical for developing targeted interventions to safeguard nurse welfare and optimize clinical outcomes.

However, existing studies have mainly focused on the prevalence of presenteeism and its influencing factors, such as work stress, burnout, job satisfaction, and organizational support. Much of the existing evidence has been generated from quantitative survey studies, which are useful for identifying associated factors but provide limited insights into nurses’ subjective experiences, contextual decision‐making processes, and perceived meanings of working while ill. In addition, previous studies have often examined nurses as a broad occupational group, while relatively limited attention has been paid to emergency nurses, who work in a highly unpredictable clinical environment characterized by rapid changes in patient conditions, high workload intensity, staffing pressure, and urgent care demands [11, 12]. Less is known about how emergency nurses experience and interpret working while ill, how they balance personal health with professional responsibility, and how staffing pressure, team expectations, and departmental support influence such decisions in daily emergency nursing practice. Therefore, this study adopted a qualitative approach to explore the experiences and influencing factors of presenteeism among emergency nurses.

The theoretical framework of presenteeism behavior, proposed by Jianmin (2015) [13], categorizes the antecedents of presenteeism into four domains: individual‐level, job‐related, organizational‐level, and team‐level factors. As the present study aimed to identify factors influencing presenteeism among emergency nurses, these four antecedent domains provided the main theoretical basis for developing the interview guide and interpreting the findings.

The study adopts Sun’s framework to conduct in‐depth interviews with ED nurses, aiming to identify contextual and systemic drivers of presenteeism. The findings are expected to provide practical evidence for nursing managers to develop supportive strategies, including more flexible staffing and rostering, nonpunitive sick‐leave practices, timely workload redistribution, team‐based support, and accessible psychological or occupational health resources.

## 2. Method

### 2.1. Participants

The study employed purposive sampling, with the sample size determined by informational saturation. To enhance the diversity and richness of the qualitative data, participants with different demographic and professional characteristics, including age, gender, educational background, years of emergency nursing experience, and professional position, were intentionally included. Recruitment continued until informational saturation was reached, defined as the point at which no substantially new themes, categories, or meanings emerged from subsequent interviews [14]. From January 01, 2025, to March 01, 2025, ED nurses from a tertiary hospital in Shanxi Province were recruited. Inclusion criteria were as follows: (1) registered emergency nurses currently in practice; (2) > 1 year of work experience; and (3) provided informed consent to participate. Exclusion criteria included the following: (1) nurses on external training programs; (2) those on extended leave; and (3) visiting nursing staff. Data were further screened based on the following: (1) response time < 4 min and (2) obviously erroneous or patterned responses. A total of 15 nurses (5 male, 10 female) were included. To ensure confidentiality, participants were anonymized as P1–P15.

### 2.2. Study Design

This study adopted a qualitative design informed by a descriptive phenomenological perspective to gain an in‐depth understanding of emergency nurses’ experiences, perceptions, and contextual determinants of presenteeism. Data were collected through semistructured, face‐to‐face, in‐depth interviews. The interview guide was informed by the theoretical framework of presenteeism behavior proposed by Sun et al. [13]. As this study focused on factors influencing presenteeism among emergency nurses, we mainly drew on the four antecedent domains of the framework: individual‐level factors, job‐related factors, organizational‐level factors, and team‐level factors. Specifically, the framework sensitized the research team to multilevel determinants, including personal health conditions, psychological resilience, workload pressure, burnout, perceived work performance, family support, collegial and team support, and institutional policies. This approach strengthened the theoretical coherence and explanatory value of the findings while allowing themes to emerge from participants’ lived experiences. The study was reported in accordance with the COREQ checklist for qualitative research [15].

#### 2.2.1. Development of the Interview Protocol

The initial semistructured interview guide was developed based on the theoretical framework of presenteeism behavior proposed by Sun et al. [13] and a review of relevant literature. The four antecedent domains of the framework—individual‐level, job‐related, organizational‐level, and team‐level factors—were used to guide the development of the preliminary interview questions.

Two preliminary interviews were conducted with emergency nurses to assess the clarity, relevance, and feasibility of the interview guide. Based on the preliminary interviews and expert feedback, the wording and sequence of several questions were refined to make them clearer and more open‐ended. The pilot interview data were not included in the final analysis.

The final interview guide included the following main questions: (1) How do you understand presenteeism among nurses? (2) Could you describe any presenteeism behavior you have experienced or witnessed among colleagues, including the physical condition and work situation at that time? (3) What do you think are the main reasons for presenteeism among emergency nurses? (4) How does working while unwell affect nurses’ health, work performance, or clinical practice? (5) Could you describe the support and understanding you received when working while sick, including where this support came from? (6) What measures do you think the hospital or department could take to reduce presenteeism among emergency nurses? and (7) Is there anything else you would like to add about presenteeism in emergency nursing practice?

#### 2.2.2. Data Collection Procedures

The principal investigator conducted one‐on‐one, face‐to‐face interviews with voluntarily participating ED nurses. The interviews were conducted by a nurse researcher with formal training in qualitative methodology and a master’s degree in nursing, who was concurrently practicing as an ED nurse, thereby facilitating the development of a trusting rapport with participants. Interviews were performed in quiet, undisturbed settings and lasted 30–40 min each. Following a predetermined interview protocol, the investigator objectively and thoroughly explored participants’ perspectives and experiences while ensuring an authentic representation of their views. All interviews were audio‐recorded with digital voice recorders and supplemented with written notes for accuracy.

#### 2.2.3. Data Analysis

Within 24 h after each interview, Y.Z.J. transcribed the audio recordings verbatim, and N.L. checked the transcripts against the original recordings for accuracy. All transcripts were anonymized using participant identifiers from P1 to P15 and imported into NVivo 8.0 for data management. Data were analyzed using Colaizzi’s seven‐step phenomenological method.

Y.Z.J. and N.L. read the transcripts repeatedly to become familiar with the data. They then independently extracted significant statements related to presenteeism, including nurses’ experiences of working while ill, perceived reasons for presenteeism, physical and psychological responses, work‐related influences, team support, and organizational factors. These statements were condensed into meaning units and then grouped into initial codes. Similar codes were further compared and organized into categories, subthemes, and themes. Theme development was informed by both the interview data and the theoretical framework of presenteeism behavior. Codes and categories were retained when they were directly related to the research aim, repeatedly reflected in participants’ accounts, or provided meaningful explanations of presenteeism in the emergency nursing context.

To enhance intercoder consistency, Y.Z.J. and N.L. independently coded the transcripts and compared their coding results. Disagreements regarding code assignment, category grouping, or theme naming were discussed with reference to the original transcripts until consensus was reached. When disagreements remained, W.J.Z. reviewed the relevant excerpts and provided methodological guidance. The final themes were agreed upon by the research team and supported by representative quotations from participants.

### 2.3. Rigor

Interviews were conducted by a qualified nurse researcher trained in qualitative methods. Prior to each session, participants were consulted to schedule interviews, which were held in private, quiet environments to ensure data integrity. Following each interview, two independent researchers transcribed the audio recordings, after which transcripts were returned to participants for verification to confirm accuracy.

### 2.4. Ethics Approval Statement

Ethical approval for this study was granted by the Ethics Committee of Shanxi Provincial People’s Hospital (2024‐Shanxi Provincial People’s Hospital Ethics Committee‐372). The Ethics Committee approved the use of verbal informed consent for participant recruitment. The study was conducted in accordance with the principles of the Declaration of Helsinki. Prior to each interview, participants were informed about the purpose of the study, interview procedures, confidentiality measures, and their right to withdraw at any time without penalty. Verbal informed consent was obtained from all participants before the interview commenced. Participants’ consent was documented through audio recording and interview records. All data were anonymized prior to analysis.

## 3. Results

### 3.1. General Characteristics of Study Participants

A total of 15 ED nurses participated in this study. The participants included 10 females and 5 males. Their ages ranged from 25 to 45 years, with a mean age of 32.8 years. Most participants held a bachelor’s degree (80.0%, 12/15), while three held a master’s degree (20.0%, 3/15). Years of emergency nursing experience ranged from 1 to 23, with a mean of 10.3. Regarding professional position, most participants were staff nurses without a formal managerial position (80.0%, 12/15), followed by team leaders (13.3%, 2/15) and one nurse manager (6.7%, 1/15). Each interview lasted 30–40 min, and the interviews yielded approximately 106,000 words of transcribed textual data. The characteristics of participants are shown in Table 1.

**TABLE 1:** The characteristics of interviewees (*n* = 15).

Number of participants	Male/female	Age (year)	Education	Work experience (years)	Position
P1	F	37	Master	14	Nurse manager
P2	F	40	Bachelor	18	None
P3	F	36	Bachelor	14	Team leader
P4	M	35	Bachelor	13	Team leader
P5	F	45	Bachelor	23	None
P6	M	34	Bachelor	12	None
P7	F	34	Bachelor	12	None
P8	M	33	Bachelor	11	None
P9	F	32	Bachelor	10	None
P10	F	30	Bachelor	8	None
P11	F	30	Master	4	None
P12	M	28	Bachelor	6	None
P13	M	27	Bachelor	5	None
P14	F	26	Master	1	None
P15	F	25	Bachelor	3	None

### 3.2. Theme 1: Personal Health

#### 3.2.1. Physical Health

Participants frequently described physical health as an important consideration when deciding whether to work while ill. Participants reported that symptom severity was an important basis for deciding whether to take sick leave. Minor symptoms were often tolerated, whereas more serious conditions prompted nurses to reconsider continuing work.

“After fainting at work led to a cardiac diagnosis, I now take leave when unwell—health must come first. (P5)

“In my career, only minor ailments like colds or styes have occurred; sick leave decisions always depend on severity. (P11)

Some participants also described self‐management strategies when symptoms were mild. Rather than immediately taking sick leave, they attempted to control symptoms and continue working when they believed their condition would not affect patient care.

“As a male nurse with physiological advantages, I proactively mitigate minor symptoms and rarely take sick leave. (P8)

#### 3.2.2. Psychological Resilience

Participants described psychological resilience as important in shaping their coping with presenteeism in a high‐pressure clinical environment. Participants described that emergency nursing requires rapid responses, emotional regulation, and the ability to remain calm in uncertain or urgent situations. Nurses with more emergency experience tended to report greater confidence in handling stressful situations, whereas less experienced nurses reported greater anxiety and uncertainty.

“Extensive experience enables composed responses to emergencies without anxiety or depressive symptoms. (P2)

“As a new graduate, inadequate technical mastery and persistent safety concerns intensify anxiety and depressive symptomatology. (P14)

Some participants described psychological tension when professional responsibility conflicted with personal health needs. In these accounts, working while ill was not only a physical burden but also a source of emotional conflict.

“A sense of duty compels me to work through illness, though this self‐sacrifice breeds psychological dissonance. (P10)

### 3.3. Theme 2: Work‐Related Factors

#### 3.3.1. Job Satisfaction

Participants described job satisfaction in relation to the work environment, team relationships, management fairness, and recognition of their work. Participants described that supportive departmental activities and positive collegial relationships helped them maintain a more positive attitude toward emergency nursing.

“Department‐organized team‐building retreats enhance physical resilience while strengthening collegial bonds, directly elevating job satisfaction. (P12)

Some participants also linked job satisfaction to fair and humanistic management. Transparent performance evaluation, reasonable sick‐leave treatment, and recognition of overtime work were perceived as important factors that helped nurses feel supported by the department.

“Though physically demanding, emergency nursing provides reciprocal fulfillment through transparent performance metrics and humanistic management practices that avoid punitive productivity measures. (P6)

“The equitable compensation framework—preserving performance ratings during justified sick leave while offering overtime remuneration—optimizes workforce flexibility without compromising care standards. (P7)

#### 3.3.2. Burnout

Participants described burnout as a common experience amid persistent occupational pressure and presenteeism. Participants reported experiencing fatigue, emotional exhaustion, and feelings of physical and psychological depletion when working while unwell. However, nurses with longer emergency nursing experience described that clinical familiarity and coping experience helped them better tolerate stressful work situations.

“Thirteen years in emergency care have instilled adaptive resilience—the acuity no longer overwhelms, and procedural mastery buffers burnout. (P4)

In contrast, less experienced nurses described greater fatigue when working while ill, partly because clinical tasks took longer and required more effort.

“Working while ill induces profound fatigue, likely compounded by limited clinical experience that prolongs task completion times. (P15)

#### 3.3.3. Performance

Work performance was affected by nurses’ physical condition, symptom severity, and perceived ability to maintain safe care. Some participants described that mild discomfort did not necessarily prevent them from completing routine nursing tasks, but it did require greater attention and self‐monitoring during work.

“When the symptoms are mild, I can usually continue working and complete routine nursing tasks, but I need to pay more attention to my physical condition and avoid making mistakes. (P5)

Other participants noted that working while unwell could reduce concentration, reaction speed, and work efficiency, particularly during busy emergency shifts. This made them more cautious when performing nursing tasks.

“When I am unwell during a busy shift, my concentration and reaction speed are affected. I can still finish the work, but it takes more effort and I worry that my efficiency may decline. (P1)

### 3.4. Theme 3: Social and Organizational Support

#### 3.4.1. Family Support

Participants described family support as playing both protective and reinforcing roles in relation to presenteeism. Participants described that family members’ understanding, reminders, and practical assistance eased psychological pressure and created opportunities for rest after work.

“When I felt unwell but still had to go to work, my family usually understood my situation. They would remind me to pay attention to my health and told me not to force myself too much. This kind of understanding reduced my psychological pressure. (P6)

“After a busy shift, especially when I was working while sick, my family would take over most of the household responsibilities. This allowed me to rest after work and recover physically. (P9)

“My family would advise me to take leave or see a doctor when my symptoms were obvious. Their concern made me realize that continuing to work while seriously unwell might not be good for myself or for patient care. (P11)

At the same time, family responsibility could also encourage some nurses to continue working despite minor illness, particularly when they were concerned about income or family disruption.

“Sometimes I still choose to work despite minor illness because I feel responsible for my family. I did not want my absence or reduced income to affect family life, so I tended to endure minor discomfort and continue working. (P13)

#### 3.4.2. Collegial and Team Support

Participants described collegial and team support as important in coping with the burden associated with presenteeism by providing emotional reassurance, task sharing, shift adjustment, and workload redistribution. Participants described that support from colleagues and head nurses made it easier to continue working when symptoms were mild and helped reduce psychological strain when they felt unwell.

“During illness, charge nurses proactively redistribute workloads in high‐demand zones to ensure patient safety while accommodating health needs. Colleagues provide both task‐sharing and emotional solidarity, which significantly diminish negative emotions. (P2)

“Leadership compassion and peer‐driven shift adjustments—such as early handovers to facilitate rest—attenuate physical discomfort and psychological strain. (P15)

Participants described team cohesion as important for maintaining workflow when nurses experienced health‐related difficulties. Participants described that strong interpersonal relationships within the department facilitated colleague coverage during unexpected illness or temporary absences.

“These cohesion‐focused interventions cultivate robust interpersonal relationships, synergistically improving workflow efficacy. Enhanced collegial support networks also provide critical coverage during health‐related absences, thereby reducing productivity losses associated with unplanned work disruptions. (P3)

#### 3.4.3. Institutional Policies

Institutional policies represented the formal organizational support available to nurses. Participants described that protected rest periods, wellness programs, psychological counseling, and health‐support resources as important forms of organizational support when working while ill.

“Departmentally allocated rest periods and wellness programs are essential for maintaining our physical and mental health, enabling better recovery of energy and cognitive resilience, which minimizes work interruptions linked to health deterioration. (P9)

“Confidential counseling channels and mental health resources in the department help us relieve stress and feel more supported when we are under pressure. (P13)

“During periods of low clinical demand, staffing levels are dynamically optimized to minimize resource redundancy, ensuring adequate rest for personnel. Concurrently, the department routinely organizes psychological workshops led by in‐house mental health experts to alleviate stress and proactively identify psychosocial concerns among nursing staff. Furthermore, periodic team‐building initiatives, including physical fitness‐oriented group activities, are implemented to promote holistic well‐being. (P1)

## 4. Discussion

### 4.1. Implications of Presenteeism on Emergency Nurses’ Personal Health

This study suggests that ED nurses experience multifaceted health challenges, and participants described physical conditions and psychological distress as important considerations related to presenteeism. Despite strong professional commitment to patient safety, working while ill exacerbates somatic symptoms and negatively affects mental health [16]. Nurses predominantly base sick‐leave decisions on illness severity, prioritizing occupational responsibilities over personal well‐being—a pattern reflecting professional dedication but posing long‐term health risks. Consistent with global evidence, our findings suggest potential relationships among emotional labor, job satisfaction, and perceived work performance [17–19]. Previous studies have suggested that greater emotional labor may be associated with increased psychological strain, detrimentally influencing both work efficacy and satisfaction. As highlighted by Hwang et al. [20], nurses’ management of emotional displays to maintain professional personas intensifies stress, thereby reducing job satisfaction and impairing performance. Furthermore, Back et al. [21] establish that heightened emotional labor in emergency nurses escalates burnout dimensions—emotional exhaustion, depersonalization, and diminished personal accomplishment. These burnout manifestations not only compromise health but also elevate turnover intentions, as chronic stress undermines occupational sustainability.

### 4.2. Impact of Presenteeism on Job Performance and Satisfaction

Participants described receiving support from family members, colleagues, and supervisors when practicing presenteeism, with such support partially alleviating physical discomfort and psychological distress. However, chronic occupational stressors, persistent occupational stressors, and experiences of presenteeism were frequently described as being associated with lower job satisfaction. While emergency nurses’ psychosomatic health is demonstrably compromised by presenteeism, the identified compensatory mechanisms—particularly family support, leadership accommodation, and collegial and team support—may mitigate acute symptom exacerbation [22, 23]. These observations align with Guo et al. [24], who established correlations between nursing presenteeism and occupational stress mediators, and Zhang et al. [25], whose work delineates interactions among emergency nurses’ workload stress, coping modalities, and self‐efficacy [26, 27].

### 4.3. Social and Organizational Support Strategies for Presenteeism

Social and organizational support strategies are critical for mitigating presenteeism among emergency nurses [28]. Our findings suggest that family support, collegial and team support, and institutional policies may jointly influence nurses’ decisions to work while ill by shaping emotional coping, workload coordination, and access to formal health‐support resources. As evidenced by Yonghui et al. [29], emergency nurses’ perceived organizational support positively correlates with job satisfaction—a factor potentially relevant to presenteeism. Furthermore, the theoretical framework of presenteeism proposed by Huan et al. [30] underscores the importance of organizational‐ and team‐level factors, a premise further extended by our findings on family support as an additional social resource. Strategic measures, including optimized shift scheduling, enhanced interdisciplinary collaboration, and systematic psychological support programs, may help reduce presenteeism by improving staffing flexibility, strengthening team coordination, and enhancing psychological support. Concurrently, individual nurse engagement in health literacy initiatives and stress resilience training is imperative to synergize institutional efforts, as sustained behavioral adaptation requires dual organizational and individual alignment [31, 32].

### 4.4. Implications for Nursing

Presenteeism among emergency nurses reflects the combined influence of individual motivation, workload pressures, and organizational culture. The findings highlight the need for nursing management and policymakers to recognize presenteeism as both a workforce well‐being concern and a patient safety issue. At the clinical level, nursing leaders should foster supportive working environments that encourage nurses to prioritize their health needs without fear of stigma or negative evaluation. Initiatives such as flexible rostering, timely staffing adjustments, regular psychological support, and collegial and team support may help reduce the burden associated with working while unwell and create a supportive work environment that enables nurses to seek help or take sick leave when necessary. Education on self‐care, resilience, and professional boundaries may further empower nurses to make healthier decisions about their work participation.

At a broader policy level, healthcare organizations and regulatory bodies should develop policies that normalize appropriate sick leave and ensure adequate staffing to accommodate illness‐related absence. Policies that emphasize employee well‐being, psychological safety, and respectful workplace culture are essential to promoting sustainable emergency nursing practice. Investment in organizational support structures, such as employee assistance services and confidential counseling, may protect both workforce stability and patient care quality. By strengthening health‐promoting work environments, nursing and health policy can help reduce presenteeism and sustain a resilient, high‐performing nursing workforce in emergency care settings.

### 4.5. Theoretical Implications

The findings of this study support the explanatory value of the theoretical framework of presenteeism behavior in the context of emergency nursing. Consistent with the four antecedent domains of this framework, presenteeism among emergency nurses was shaped by multiple interacting factors rather than by individual health status alone. Personal health factors, including physical illness and psychological resilience, reflected individual‐level influences on nurses’ decisions to work while ill. Work‐related factors, such as job satisfaction, burnout, and perceived work performance, represented job‐level conditions that shaped presenteeism experiences. Social and organizational support, including family support, collegial and team support, and institutional policies, reflected interpersonal, team‐level, and organizational resources that could either buffer or reinforce presenteeism behavior.

This study further illustrates the applicability of the theoretical framework by showing that the four antecedent domains are closely interconnected in the emergency nursing context. For example, physical discomfort and psychological resilience interacted with workload pressure, professional responsibility, staffing arrangements, and team relationships. These findings suggest that presenteeism should not be understood simply as an individual health‐related behavior but as a multilevel occupational phenomenon embedded in the emergency care environment.

Therefore, the present study contributes to the theoretical understanding of nurse presenteeism by linking individual‐level, job‐related, organizational‐level, and team‐level factors within a unified explanatory framework. Future research should further examine how these multilevel factors interact across different hospital settings, regions, and nursing specialties.

## 5. Limitations

This study has several limitations that should be considered when interpreting the findings. First, participants were recruited from a single tertiary hospital in Shanxi Province using purposive sampling, which may limit the transferability of the findings to emergency nurses working in other regions or in hospitals of different levels. Although this qualitative study aimed to obtain an in‐depth understanding of emergency nurses’ experiences rather than statistical representativeness, and recruitment continued until informational saturation was reached, the institutional and regional context should still be considered when interpreting the findings. Future research should include larger samples from multiple centers, different hospital levels, and diverse geographical regions to further validate and extend these findings. Comparative studies across different healthcare settings may also help to identify contextual variations in presenteeism among emergency nurses. Second, as a qualitative study based on self‐reported data, the findings are susceptible to social desirability bias, in which participants may have provided responses they believed to be socially acceptable rather than fully reflecting their actual experiences and behaviors. Additionally, the cross‐sectional nature of the interviews captures perceptions at a single point in time and cannot establish causal relationships among the identified factors. Future research would benefit from employing multicenter, longitudinal, or mixed‐methods designs to validate and expand upon these findings.

## 6. Conclusion

The findings suggest that ED nurses demonstrate strong professional commitment and context‐specific adaptive strategies when experiencing presenteeism. Evidence‐informed multilevel interventions that combine individual behavioral engagement, social support, collegial and team support, and organizational policies may help address presenteeism and support the nursing workforce well‐being. These coordinated approaches may support staff well‐being and create conditions that promote high‐quality, safe patient care.

## Author Contributions

Study conception and design and manuscript drafting: Zhuojing Yang, Li Niu, and Yuyan Zhao.

Data collection: Xiaoyi Cao and Lili Wang.

Data analysis: Xiaoyi Cao and Qian Zhao.

Study supervision, critical revisions for important intellectual content, and administrative and technical support: Juzi Wang.

Material support: Lili Wang, Qian Zhao, and Juzi Wang.

## Funding

This work was supported by the Scientific Research Project of Shanxi Provincial Health Commission Scientific Research Project (Grant Number 2024040), Major Research Program on High‐Quality Human Resources Development of Shanxi Province (Grant Number SXRLZYZD2539), and Shanxi Nursing Association Research Project (Grant Number SXHLKY‐202502).

## Conflicts of Interest

The authors declare no conflicts of interest.

## Data Availability

The data that support the findings of this study are available on request from the corresponding author. The data are not publicly available due to privacy or ethical restrictions.

## References

[1] Lynch A. , Britt T. W. , Shuffler M. , McCallus R. , and Hirsh E. , The Impact of Work Demands and Meaningful Work on the Burnout and Mental Health Strain of Emergency Medicine Clinicians, Stress and Health. (2026) 42, no. 2, 10.1002/smi.70160. PMC12980698 41817114

[2] Mu X. , Baldonedo-Mosteiro M. , González M. B. , Baldonedo-Cernuda R. , Franco-Correia S. , and Mosteiro-Díaz M. P. , Psychological Distress and Presenteeism During the Pandemic Period: A Quantitative Study Among Nurses From Emergency and Critical Care, Journal of Nursing Management. (2026) 2026, no. 1, 10.1155/jonm/4278999. PMC13088158 41995116

[3] Baier N. , Roth K. , Felgner S. , and Henschke C. , Burnout and Safety Outcomes–a Cross-Sectional Nationwide Survey of EMS-Workers in Germany, BMC Emergency Medicine. (2018) 18, no. 1, 10.1186/s12873-018-0177-2. PMC6102842 30126358

[4] Leszczyński P. , Panczyk M. , Podgórski M. et al., Determinants of Occupational Burnout Among Employees of the Emergency Medical Services in Poland, Annals of Agricultural and Environmental Medicine. (2019) 26, no. 1, 114–119, 10.26444/aaem/94294. 30922040

[5] Kinman G. , Sickness Presenteeism at Work: Prevalence, Costs and Management, British Medical Bulletin. (2019) 129, no. 1, 69–78, 10.1093/bmb/ldy043. 30649219

[6] Yang T. , Liu R. , and Deng J. , Does Co-Worker Presenteeism Increase Innovative Behavior? Evidence From IT Professionals Under the 996 Work Regime in China, Frontiers in Psychology. (2021) 12, 10.3389/fpsyg.2021.681505. PMC8281301 34276503

[7] Ferreira A. I. , Mach M. , Martinez L. F. , and Miraglia M. , Sickness Presenteeism in the Aftermath of COVID-19: Is Presenteeism Remote-Work Behavior the New (Ab)Normal?, Frontiers in Psychology. (2021) 12, 10.3389/fpsyg.2021.748053. PMC8830031 35153891

[8] Freeling M. , Rainbow J. G. , and Chamberlain D. , Painting a Picture of Nurse Presenteeism: A Multi-Country Integrative Review, International Journal of Nursing Studies. (2020) 109, 10.1016/j.ijnurstu.2020.103659. 32585449

[9] Mohammadi M. M. , Nayeri N. D. , Varaei S. , and Rasti A. , The Nurse Without a Nurse: The Antecedents of Presenteeism in Nursing, BMC Nursing. (2021) 20, no. 1, 10.1186/s12912-021-00669-1. PMC8361635 34389006

[10] Li J. , Zhang Q. , Duan Y. , Huang M. , and Guo M. , Emotional Support, Emotional Exhaustion, Mental Health, and Turnover Intention Among China Nurses in Emergency Departments: A Cross-Sectional Survey, Journal of Nursing Research. (2025) 33, no. 6, 10.1097/jnr.0000000000000710. PMC12662123 41292088

[11] Vizer L. M. , Liu C. C. , Kwong E. K. et al., Workplace Stressors Associated With Burnout Among Emergency Nurses and Other Emergency Healthcare Professionals: A Convergent Parallel Approach With a Multilevel Design, Journal of Nursing Management. (2026) 2026, no. 1, 10.1155/jonm/7360364. PMC13201899 42186289

[12] Salvarani V. , Rampoldi G. , Ardenghi S. et al., Protecting Emergency Room Nurses From Burnout: The Role of Dispositional Mindfulness, Emotion Regulation and Empathy, Journal of Nursing Management. (2019) 27, no. 4, 765–774, 10.1111/jonm.12771. 30887587

[13] Jianmin S. and Yejun Z. , Presenteeism in the Workplace: A New Topic in Organization and Management Research, Advances in Psychological Science. (2015) 23, no. 04, 654–668.

[14] Kerr C. , Nixon A. , and Wild D. , Assessing and Demonstrating Data Saturation in Qualitative Inquiry Supporting Patient-Reported Outcomes Research, Expert Review of Pharmacoeconomics & Outcomes Research. (2010) 10, no. 3, 269–281, 10.1586/erp.10.30. 20545592

[15] Tong A. , Sainsbury P. , and Craig J. , Consolidated Criteria for Reporting Qualitative Research (COREQ): A 32-Item Checklist for Interviews and Focus Groups, International Journal for Quality in Health Care. (2007) 19, no. 6, 349–357, 10.1093/intqhc/mzm042. 17872937

[16] Moore A. and Knutsen Glette M. , Nurses’ Experience With Presenteeism and the Potential Consequences on Patient Safety: A Qualitative Study Among Nurses at Out-of-Hours Emergency Primary Care Facilities, BMJ Open. (2023) 13, no. 11, 10.1136/bmjopen-2023-076136. PMC10668197 37989382

[17] Roth K. , Köppen J. , and Henschke C. , Workplace Violence and Burnout Among Emergency Medical Service Workers and Nurses in Germany: A Cross-Sectional Study, Human Resources for Health. (2025) 23, no. 1, 10.1186/s12960-025-01026-y. PMC12632002 41267025

[18] Abbaszadeh R. , Ahmadi F. , Khoobi M. , Kazemnejad A. , and Vaismoradi M. , A Scoping Review of Fatigue Among Nurses in Critical Care Units, Nursing in Critical Care. (2025) 30, no. 4, 10.1111/nicc.70088. 40635546

[19] Huang Y. , Wang J. , Ni W. et al., The Status of Presenteeism Among Nurses: A Latent Profile Analysis, Journal of Advanced Nursing. (2025) 82, no. 7, 7325–7336, 10.1111/jan.70375. 41217068

[20] Hwang W. J. and Park E. H. , Developing a Structural Equation Model From Grandey’s Emotional Regulation Model to Measure Nurses’ Emotional Labor, Job Satisfaction, and Job Performance, Applied Nursing Research. (2022) 64, 10.1016/j.apnr.2021.151557. 35307133

[21] Back C. Y. , Hyun D. S. , and Chang S. J. , [Association Between Emotional Labor, Emotional Dissonance, Burnout and Turnover Intention in Clinical Nurses: A Multiple-Group Path Analysis Across Job Satisfaction], Journal of Korean Academy of Nursing. (2017) 47, no. 6, 770–780, 10.4040/jkan.2017.47.6.770. 29326408

[22] Kazemi S. , Gholizadeh M. , Rajaee M. , Yousefnezhad M. , and Kakhki S. K. , Presenteeism, Moral Courage, and Moral Disengagement Among Nurses: A Cross-Sectional Structural Equation Modeling Study, BMC Nursing. (2025) 24, no. 1, 10.1186/s12912-025-03779-2. PMC12392522 40877863

[23] Achmet V. and Lauzier M. , Toward a Better Understanding of Emotional Exhaustion Among First-Line Nurse Managers: The Role of Psychological Stress, Presenteeism, and Leader Loyalty, Journal of Nursing Management. (2025) 2025, no. 1, 10.1155/jonm/8865098. PMC12685427 41368334

[24] Back C. Y. , Hyun D. S. , Jeung D. Y. , and Chang S. J. , Mediating Effects of Burnout in the Association Between Emotional Labor and Turnover Intention in Korean Clinical Nurses, Safety and Health at Work. (2020) 11, no. 1, 88–96, 10.1016/j.shaw.2020.01.002. 32206378 PMC7078559

[25] Ruxin J. , Hui Z. , Jihao Z. , Yongxin L. , and Shujie G. , Study on Correlation Between Nurses’ Presenteeism Behaviour and Mental Health Status and Turnover Intention, Chinese Nursing Research. (2023) 37, no. 02, 327–332.

[26] Andres E. B. , Lui J. N. M. , Song W. , and Johnston J. M. , Exploring Hong Kong Nurses’ Decision-Making Processes Around Presenteeism, Occupational Medicine (London). (2021) 71, no. 4-5, 189–195, 10.1093/occmed/kqab047. 33963871

[27] Atalla A. D. G. , El-Ashry A. M. , and Mohamed S. M. S. , The Influence of Job Enrichment on Job Embeddedness and Sick Presenteeism Among Nurses, International Nursing Review. (2025) 72, no. 2, 10.1111/inr.13043. 39359147

[28] Hou H. , Jiang Y. , Xia C. et al., The Lived Experience of Presenteeism Among Emergency Nurses: A Qualitative Study, BMC Nursing. (2025) 24, no. 1, 10.1186/s12912-025-03902-3. PMC12512700 41068708

[29] Yonghui C. , Wei Z. , and Cai W. , Study on Correlation Between Work Pressure and Job Burnout of Nurses in Emergency Department in a Third Grade A Hospital, Chinese Nursing Research. (2015) 29, no. 27, 3402–3404.

[30] Huan L. , Xin L. , Ning Z. et al., The Effect of Constructing Nursing Team Support Strategy on Job Satisfaction of Nurses in Emergency Department, Chinese Hospitals. (2018) 22, no. 08, 68–70, 10.19660/j.issn.1671-0592.2018.08.22.

[31] Huang H. , Li F. , and Jiang Y. , Connor Davidson Resilience Scores, Perceived Organizational Support and Workplace Violence Among Emergency Nurses, International Emergency Nursing. (2024) 75, 10.1016/j.ienj.2024.101489. 38986269

[32] Alsalmi M. and Alilyyani B. , The Role of Authentic Leadership in Nurses’ Stress and Burnout in Emergency Departments, Leadership in Health Services (Bradf Engl).(2023) 37, no. 1, 147–158, 10.1108/lhs-01-2023-0005. 37606378

